# Machine Translation Utilizing the Frequent-Item Set Concept

**DOI:** 10.3390/s21041493

**Published:** 2021-02-21

**Authors:** Hanan A. Hosni Mahmoud, Hanan Abdullah Mengash

**Affiliations:** 1 Department of Computer Sciences, College of Computer and Information Sciences, Princess Nourah bint Abdulrahman University, Riyadh P.O. Box 11671, Saudi Arabia; HAhosni@pnu.edu.sa; 2 Department of Information Systems, College of Computer and Information Sciences, Princess Nourah bint Abdulrahman University, Riyadh P.O. Box 11671, Saudi Arabia

**Keywords:** machine translation, frequent-item set, bilingual corpus, BLEU score

## Abstract

In this paper, we introduce new concepts in the machine translation paradigm. We treat the corpus as a database of frequent word sets. A translation request triggers association rules joining phrases present in the source language, and phrases present in the target language. It has to be noted that a sequential scan of the corpus for such phrases will increase the response time in an unexpected manner. We introduce the pre-processing of the bilingual corpus through proposing a data structure called Corpus-Trie (CT) that renders a bilingual parallel corpus in a compact data structure representing frequent data items sets. We also present algorithms which utilize the CT to respond to translation requests and explore novel techniques in exhaustive experiments. Experiments were performed on specific language pairs, although the proposed method is not restricted to any specific language. Moreover, the proposed Corpus-Trie can be extended from bilingual corpora to accommodate multi-language corpora. Experiments indicated that the response time of a translation request is logarithmic to the count of unrepeated phrases in the original bilingual corpus (and thus, the Corpus-Trie size). In practical situations, 5–20% of the log of the number of the nodes have to be visited. The experimental results indicate that the BLEU score for the proposed CT system increases with the size of the number of phrases in the CT, for both English-Arabic and English-French translations. The proposed CT system was demonstrated to be better than both Omega-T and Apertium in quality of translation from a corpus size exceeding 1,600,000 phrases for English-Arabic translation, and 300,000 phrases for English-French translation.

## 1. Introduction

Machine Translation (MT) is an automated procedure of bilingual or multi-lingual translation [1]. There are several approaches to MT: linguistic (morphological), non-linguistic, and hybrid. Recently, statistical machine translation (SMT) and Neural Machine Translation (NMT) systems have been the leading machine translation paradigms [1,2,3]. Standard SMT techniques do not depend on any linguistic information, and do not apply any pre-processing procedures to generate the translation [4,5].

To face the challenges of machine translation, both pre-processing and post processing are utilized. Pre-processing can be accomplished via a number of lexical, syntactic, and semantic techniques. Lexical techniques include tokenization and normalization [6,7,8]. Syntactic pre-processing includes integrating additional linguistic information [9,10]. The used of named-entity notions is an example of semantic pre-processing. Pre-processing approaches are language-neutral and can be extended to other language pairs [9,10].

In this paper, we augment machine translation by using association mining concepts. Association mining can be utilized to develop efficient algorithms to analyze frequent sets representing a parallel linguistic corpus. As an analogy, it can be understood as a list of items, as in the original Apriori algorithm [11]. Co-occurring items are defined as item sets. The “support” of such an item set specifies how many groups are held in the item set. If *k* market baskets contain [X1, X2, X3], the item set’s support is k. The same analogy can be implemented where sentences are the “market baskets” and occurring ordered sets of words are the item sets. For instance, if *M* sentences contain [today is sunny], then the item set’s support is *M*. Also, if *M* sentences contain [today is sunny], and their translation in language *L* is *X*, with support *k*, a rule can be created that “today is sunny” → *X* with support *k*.

In this paper, we introduce a new concept in translation statistical association rules. We treat words as item sets, and formulate rules based on frequent-set concepts. We formulate phrases in the corpus language as trie data structures. The whole corpus is built as a trie of the tries of phrases in the Corpus-Trie. From these tries, we can induce translation from language 1 → language 2 depending on the frequency of occurrences. We devised an intelligent search technique that is linear in depth (*D*) of the CT. In this technique, there is no need to know anything in advance about the linguistic structure or grammatical rules of the languages. This methodology can be applied to any pair of languages; experiments here are in translation from English to Arabic, and from English to French.

One of the main contributions of our work is the building of the Corpus-Trie as a preprocessing step that is done offline. The translation process is the transformed into a search process in the Corpus trie, which will be a fast procedure, has a time complexity in the order of log the depth of the Corpus trie. Our concern is a preprocessing step to build a data structure based on association mining to enhance response time.

This paper presents a background and literature survey in Section 2. Section 3 presents the proposed system and a novel algorithm that can create a Corpus-Trie from a set of phrases. In Section 4, we propose the new concept of formulating a frequent-set trie representation and develop the notion of a Corpus-Trie. Section 5 introduces the experiments, demonstrating the computation time required to perform a typical translation request using the technique. It also investigates the costs of building a Corpus-Trie from a bilingual corpus. Conclusions are drawn in Section 6 with some ideas for future research.

## 2. Background and Literature Survey

We are concerned with the statistical machine translation paradigm, the association rule paradigm, and the research comparing them.

### 2.1. Statistical-Based Machine Translation (SMT)

SMT systems require huge text corpora to extract linguistic rules based on entropy [3,4,6]. SMT utilizes high-volume parallel corpora between source and destination languages, which are to a large extent available [7]. SMT proceeds from the assumption that every phrase in *T* (the target language) is a translation of a phrase in *S* (the source language) via a probabilistic formula. All pure SMT systems derive data from corpora that they have previously analyzed, and do not rely on linguistic information. SMT methods select the best representation. One of the crucial issues in SMT is the alignment problem. Alignment between phrases of the source and target languages has to be established. SMT relies on the use of statistics to solve the alignment problem and the induction of grammatical units [12,13,14,15,16].

### 2.2. Association Mining

Association rule formulation is a very effective technique which uses a data-mining paradigm to find patterns within large amounts of data [11,17,18,19]. This technique makes it feasible to find associations among different data items in large databases. We imported ideas from statistical machine translation to achieve a faster response. Association rules are generated from the dataset and reinforced by the support and confidence metrics [11]. In [17], the authors discuss the drawbacks of frequent item-set mining (FIM), such as high time complexity and high memory cost. They put forth an Array Prefix-Tree structure, which circumvents the need for recursion. They also present parallel data mining using systolic arrays. Work [18] presents parallel mining map-reduce. In [20], the authors utilize YAFIM, a parallel Apriori algorithm for memory-based mining. An incremental item-set tree for data mining on data streams is introduced in [12]. Data mining techniques have rarely been combined with SMT. In [21], for example, the authors combine data mining techniques to distinguish cases of multiple parsing in machine translation of Indian languages. They introduce knowledge-based MT by utilizing text-mining techniques. They also introduced a sub-word augmented technique for derived sub-word level representations for text comprehension. Some authors devised parallel-sentence mining from a bilingual corpus, but not with a pure Statistical machine translation model. Source-target mapping of streaming data for MT was proposed using a variety of data mining [22,23,24].

### 2.3. Machine Translation and Data Mining

In [25], the author utilizes a machine learning approach to detect stack size, which is best for beam threshold runtime values for machine translation. In [26], the authors propose a sentiment analysis approach for MT. They analyze a machine-translated Bengali corpus to its original form and induce classifiers for translation. They focus on aspect-based sentiment analysis with emphasis on the Bangla language. In [27], the authors studied predictive data analytics and introduce a new concept, namely radius of neighbors, which was found to perform better than K-nearest neighbors in translation accuracy prediction. Work [28] introduces a knowledge-based machine translation system. It utilizes text mining by identifying semantic relations among entities present in text documents. In [29], the authors introduce a systematic approach for NMT and its application to context vectors. In [30], the authors present a graph-based approach for statistical translation.

### 2.4. Neural Machine Translation (NMT)

In [28], the authors present a framework that incorporates SMT word knowledge into NMT to address word-level obstacles. The NMT decoder performed accurate word prediction in both training and testing phases utilizing SMT. In [31], the authors use phrase-based SMT to calculate the cost of phrase-based decoding of NMT output and re-rank the *n*-best outputs. In [32], the authors survey parallel corpora and collect bilingual corpora; many corpora have 100,000 parallel sentences per language pair. Many papers discuss NMT, emphasizing zero shot neural machine [31,32,33] techniques. The authors of [34] note that NMT requires smaller data sizes, as small as a few thousand training sentences. In [35], an extensive survey for low resource NMT is introduced. Also in [36], the authors describe an analytical study and evaluation methods for multilingual machine translation as well as analytic evaluation matrices of machine translation.

### 2.5. Example-Based Machine Translation (EBMT)

The authors in [37] introduced the definition of the example used in EBMT. The main issues in EBMT are example acquisition, and base management. Also, it includes the notion of target sentence generation. EBMT adopted the concept of translation by analogy via example translations, which is the main core of the EMMT training process [38].

Example of a bilingual corpus:
**English****Arabic**How much is that blue umbrella?ما هو سعر الشمسية الزرقاء ؟How much is that small bag?ما هو سعر الحقيبة الصغيرة ؟

EBMT undergoes training using bilingual parallel corpora, which include sentence pairs. The example above shows a *minimal pair example*, where the sentences differ by only one element. These sentences make it simple to learn translations of sub-sentential units.

An EBMT will learn three aspects from those sentences in the bilingual corpora:

- “How much is that X?” Corresponds to “ما هو سعر س؟”
- “Red umbrella” corresponds to “الشمسية الزرقاء”
- “Small camera” corresponds to “الحقيبة الصغيرة”

The concepts in EBMT include training process and learning from example, which is different than our approach that does not include training or learning process. Our approach converts a bilingual corpus into a compact data structure namely the Corpus Trie, and converts the machine translation process into a search in association rules like mining.

### 2.6. Critique of Existing SMT Techniques

The response time for translation requests is crucial, especially for large requests; it is also a problem for real-time translation [39]. In spite of the presence of parallel corpora with alignment already annotated [40,41], searching this database to extract a phrase and the corresponding highest-probability translation associated with it requires scanning the corpus sequentially. The corpus may include millions of sentences. Response time can be reduced by reducing the corpus volume, but this will also reduce the accuracy of translation. Researchers have proposed several algorithms to expedite response time. For example, in [6], the authors introduce concept-formation techniques that group interrelated words, which can be helpful to reduce the complexity and time of association mining [42,43].

We propose a novel technique to represent the whole parallel corpus as a trie with frequencies attached to its edges. We store the corpus representation as tries which connect phrases from the source language and translated phrases from the target language, and store different translations and their frequencies. The space required for the trie is much less than the actual corpus (because repeated phrases will be stored once along with the highest-probability translated phrases from the target language), and the response time for any translation will be enhanced extensively, as the corpus will not need to be searched sequentially. Instead, the trie will be searched, and the time complexity will be in order with the trie’s depth.

In this paper, we service the user’s wish to utilize a bilingual corpus by submitting a translation request including the phrase Ph_S_ in the source language. We treat the corpus as a database of frequent phrase sets, and assume that the user will constrain the search to phrases that include the ordered words in Ph_S_. A translation request may seek all frequent phrases containing word 1 and word 2, in order. In such cases, repeated search for all phrases would increase the response time in an unexpected manner. Therefore, in this research we emphasize pre-processing the corpus and inducing the trie representing it. We propose a data structure called Corpus-Trie and present novel algorithms that use CT to respond to translation requests, after exhaustive experiments.

## 3. Proposed Methodology

In this section, we introduce the proposed concept in translation statistical utilizing association rules. We treat words as item sets, and formulate rules based on frequent-set concepts. We formulate phrases in the corpus language as trie data structures. The whole corpus is built as a trie of the tries of phrases in the Corpus-Trie. From these tries, we can induce translation from language 1 → language 2 depending on the frequency of occurrences.

**Figure 1:**
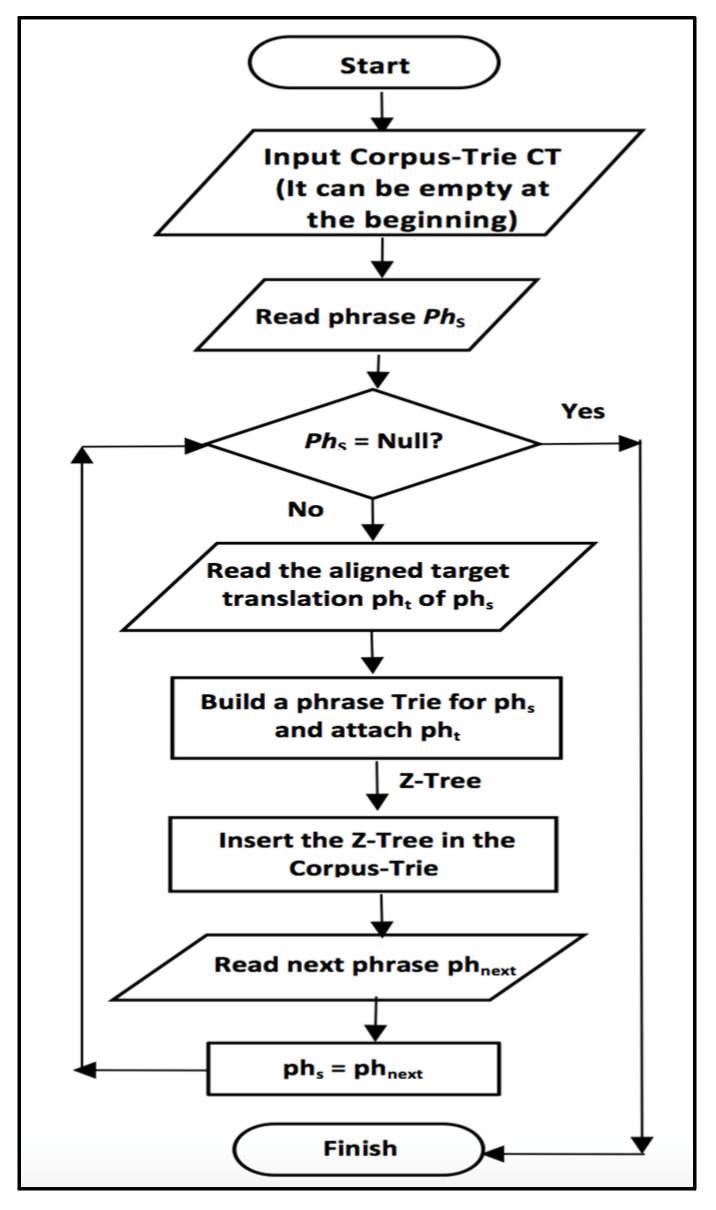
Flowchart of the CT system.

We present the proposed CT system in Figure 1 clarifying the building blocks of the system. We start by building the Corpus Trie by reading new phrases with their translations and insert them in the CT if they do not exist before. Each phrase in the source language is represented as a trie and inserted into the CT in the appropriate position (if a subset of this phrase already exists in the CT). Each phrase in the source language will be associated with multiple translations from the target language; these multiple translation will be stored in a tree-structure namely the Z-tree. The CT building process should be done offline since it is a lengthy process, as indicated in the experimental results in Section 5.

### 3.1. Building a Phrase Trie

We also introduce several algorithms to build the phrase trie-like structure as shown in Algorithm 1 in Section 3.1. Z-tree insertion is introduced in Algorithm 2, which describes the Z-tree insertion of a new translation in the Z-tree. The Z-tree is a two-level tree and is defined as having a root node and leaf nodes. Leaves are defined as an ordered pair: <content, frequency>. All the translations and their frequencies of content (parent) are added as a tree.

**Algorithm 1.** Build a phrase trie (*Input: phrase Ph, length(Ph) n; Output: Trie T*_P_).
**Start**

*// phrase Ph consists of an ordered tuple of n words: wi*
∀
*i = 1 to n*
*//T*_P_ is emptyInsert *w1* as the root node of the tree *T*_P_Parent = root;**For***i* = 1 to *n*
*wi +1= wi + wi + 1;*

*Add a left child node: N_left_ to parent;*

*Content (N_left_) = wi + 1;*
*Parent = N*_left_;
*Get all translation of the content (parent) from the target language T*

/* Translation from the Target language T will be obtained using statistical translation machine or neural machine translation machine */

*Save them to the array S_Z_*

**For**
*j = 1 to size of (S_Z_)*

*Call the procedure Z-Tree-insertion (j, S_Z_ [j])*

**End for**

**End for**

**End**


**Algorithm 2.** Z-Tree-insertion (*input j*, *S_Z_ [j]*).
**Start**
*i* = 1;Step 1:**If** node [*j*] = empty
**Then**
{Insert *S_Z_ [j] at* node [*j*]*;**Frequency (j) = Frequency (j) + 1;*}**Else if** (*S_Z_ [j] = content* (node [*j*]))
**Then**

*Frequency (i) = Frequency (i) + 1;*
**Else if** (*S_Z_ [j] < content* (node [*j*]))
**Then**
{*j = j + 1*;Go to step 1;}**Else if** (*S_Z_ [j] > content* (node [*j*]))
**Then**
{Insert a new empty node, node [*x*] between nodes: node [*j*] and node [*j + 1*];Content (node [*x*]) = *S_Z_ [j];**Frequency* (node[*x*]) = *1;**j = j + 1*;Go to step 1}
**End if**

**End**


### 3.2. Building the Corpus-Trie

We will use Algorithm 3 to build the Corpus-Trie. In order to facilitate the algorithm, we will define a phrase as a prefix of another prefix in Definition 1.

**Definition** **1.** ***Ph_S1_ is a prefix of Ph_S2_:** The symbols Ph_S1_ and Ph_S2_ denote phrases, and both of them are an ordered tuple. Ph_S1_ is a prefix of Ph_S2_ iff Ph_S1_ = [w1, w2, …, wm], Ph_S2_ = [w1, w2, …, wn], where m ≤ n. The algorithm that builds the Corpus-Trie is an incremental algorithm built through a sequence of phrase insertions. The Corpus-Trie itself is built through a single pass of the database.*

**Algorithm 3.** Build *Corpus-Trie* CT (*input Ph*_S_ = {*Ph*_S1_, *Ph*_S2_, …, *Ph_Si_*, *Ph*_Si+1_, ….., *Ph*_Sn_)./* Consider the parallel corpus. In the source language the corpus consists of sub phrase as follows:*Ph*_S_ = {*Ph*_S1_, *Ph*_S2_, ……., *Ph*_Si_, *Ph*_Si+1_, ….., *Ph*_Sn_) */
**Start**
Have a root node *n*0 = $\hat{R}$ which is an empty phraseRead *Ph*_S1_*n*1 = *n*0.childContent (*n*1) = *Ph*_S1_**For***i* = 1 to *n*Read *Ph*_Si+1_**If***Ph*_Si_ is a prefix of *Ph*_Si+1_
**Then**
{*Append*
*n*_i+1_ to be a node child to *n*_i_*Content (n*_i+1_) = *Ph*_Si+1_}
**Else**
{*Append*
*n*_i+1_ to be a node child to *n*_i−1_*Content (n*_i+1_*)* = *Ph*_Si+1_}
**End if**

**End for**

**End**


Adding a phrase to the Corpus-Trie is clarified in Example 1.

**Figure 2:**
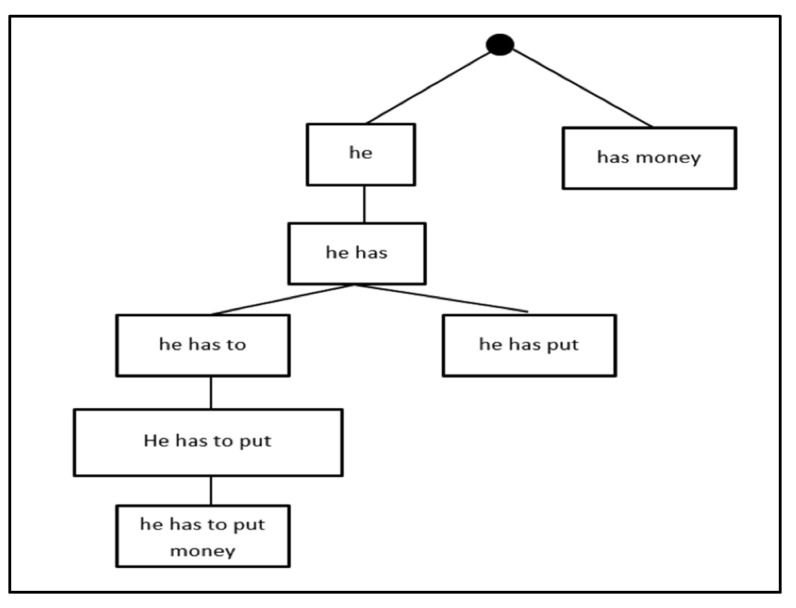
The CT illustration of Example 1.

**Example** **1.** *Figure 2 shows the trace of Algorithm 1 processing the following phrases from a bilingual corpus: Ph = {[he], [has, money], [he, has], [he, has, to], [he, has, put], [he, has, to, put, money], [ he, has, to]}.*

The following steps are performed:

1. An empty node is defined as the root node (node 0);
2. The first phrase [he] is defined as an ancestor to the root node (left child);
3. The second phrases, [has, money] and [he], are not identical. They have an empty subset. Therefore, a new child node is created and added as the right node because “has” is alphabetically greater than “he”;
4. The third phrases, [he, put] and [has, money], are not identical, as they have an empty subset. So recursively we will compare the third phrase, [he, put] and [he] are not identical, but have [he] as a subset and [he] is a prefix of [he, put]. Therefore, a left child node is created to the parent node [he];
5. The fourth phrase, [he, has, to] has a common subset with [he, has], and [he, has] is a prefix of [he, has, to], so it will be added as a left child node to [he, has]. This is added to the left child because [he, has] is a leaf node (node 4);
6. The fifth phrase, [he, has, put] has a common subset with [he, has], and [he, has] is a prefix of [he, has, put], so it will be added as a left child node to [he, has]; left child, because [he, has] is a leaf node;
7. The sixth phrase, [he, has, to] has a common subset with [he, has], and [he, has] is a prefix of [he, has, to], so it will be added as a right child node to [he, has];
8. The seventh phrase, [he, has, to, put, money] has a common subset with [he, has, to], and [he, has, to] is a prefix of [he, has, put], so it will be added as a left child node to [he, has, to]; left child because [he, has, to] is a leaf node;
9. The eighth phrase, [he, has, to] is identical to node 4 so it will not be added.

**Lemma** **1.** ***(Corpus-Trie size):** Let CT denote the Corpus-Trie built from a bilingual corpus of N distinct phrases of an average of m words per phrase, using Algorithm 1:*

*1.*  *The upper bound of the nodes’ count in the CT is m* × *N + 1;*
*2.*  *The upper bound of the number of layers in the CT is m* × *N+ 1 (worst case).*

**Proof** **(from** **definition).** The memory required to store the Corpus-Trie is much less than the size of the bilingual corpus, because it stores the repeated phrases just once, and stores only the phrase translations with the highest frequencies. Therefore, the methodology does not depict an overhead in real-world domains. □

**Figure 3:**
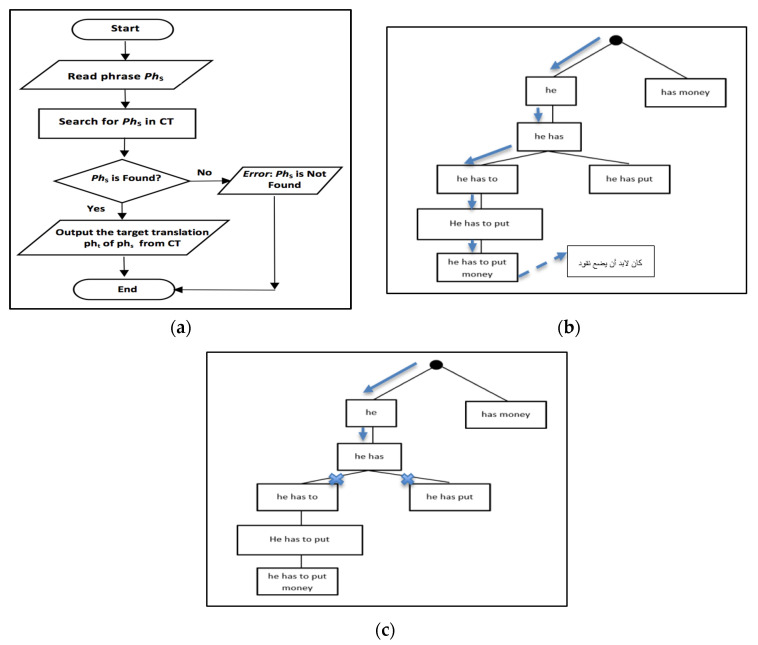
(**a**) Flowchart of the translation process; (**b**) An example showing the translation of the sentence “he has to put money”; (**c**) An example showing the translation of non-existent sentence in the Corpus Trie “he has money”.

In Figure 3a, we show a flowchart of the translation process, which mimics a search problem in the Corpus Trie, Algorithm 4 provides more details of the translation problem.

Algorithm 4 is the search algorithm, the translation process using the Corpus-Trie. It starts with the input phrase S from the source language, and divides it into words w1, w2, w3, ……wm. The word wi (i = 1 to m) is searched in the trie starting from the root of the Corpus-Trie until it is located, then the next word in S is searched, and so on continuing down the trie, if found.

Figure 3b, presents an example showing the translation of the sentence “he has to put money”, from the Corpus Trie shown previously in Figure 2. Another example of trying to translate a phrase that is not found in the Corpus Trie is shown in Figure 3c.
**Algorithm 4.** Get Translation (input key: *Ph*_S_, output node: Node).**Start****For***I = 1 to N* // *N* is number of words *w*(*i*) in key:**If***w*(*i*) *in node.children*:*node* = *node.children* [*w*(*i*)]**Else:**Return *None***End if****End For**Return *node.Ph*_T_**End**

## 4. The Notion of a Corpus-Trie

In this section, we propose a formulation for parallel corpora, without any emphasis on particular languages. The defining trait of a bilingual corpus is translation between two languages: the source language *S* and the target language *T*. Word alignment should be part of the corpus. Word alignment is done on biphrases, by our algorithm, using a bilingual dictionary to align each word in the sentence in the source language to its match in the target language.

**Figure 4:**
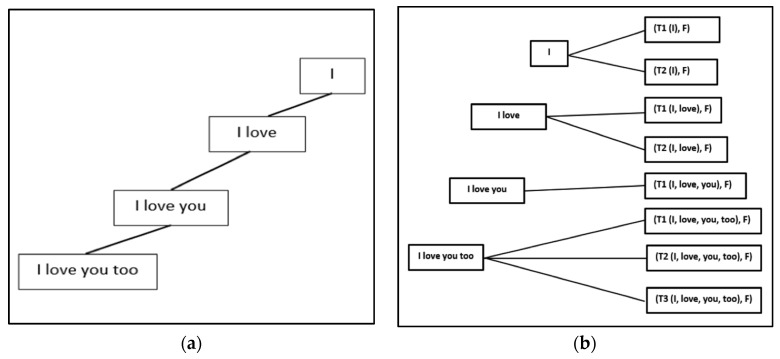
(**a**) Trie-Like structure of a phrase Ph is shown for a Phrase Ph of four words <I, love, you, too>; (**b**) Phrase Ph that includes Ph_S_ (phrase of the source language) and the corresponding Ph_T_ (phrase in the target language).

Later we will extend the formulation to “source language ⇒ multi-target language” translation. To formulate our technique, we define a translated phrase formally and recursively as a prefix-trie structure. We also introduce formulated definitions for a corpus and a Corpus-Trie in Definitions 2, 3, and 4. In addition, we describe a data structure called a Z-tree in Definition 5. Also, in Lemma 2, we prove that a phrase is a prefix trie-like structure. In Figure 4a, the trie-like structure of a phrase *Ph* is shown for a phrase *Ph* of four words < *w1*, *w2*, *w3*, *w4* >. In Definition 5, we define an extended version of phrase *Ph* to include *Ph*_S_ (phrase of the source language) and the corresponding *Ph*_T_ and *AL*_S:T_ (phrase in the target language) as depicted in Figure 4b.

**Definition** **2.** *A translated phrase Ph:*

*Define Ph as an ordered tuple Ph = <Ph_S_, <Ph_T_, AL_S:T_ >>, where:*

*2.*  *Ph_S_ and Ph_T_ are two phrases from source language and target language, respectively; Ph_T_ is the corresponding phrase translation from the target language, with alignment AL_S:T_ between phrases Ph_S_ and Ph_T_;*
*2.*  *M is the set of all words constituting Ph_S_. All words in Ph_S_ have an order-by-word location in ascending order. For any two words, the ith and jth words are ordered as i < j, i.e., w_i_ < w_j_, where w_i_ represents the word in position I;*
*3.*  *P_S_ is itself defined as Ph, which yields a recursive definition of Ph;*
*4.*  *Ph can be represented as a trie, Ph-trie (see proof in Lemma 2).*

**Definition** **3.** *The relation between Ph, Ph_S_, Ph_T_ and AL*

*1.*  *Ph_S_ is a recursive definition of other phrases as follows: P_S_ = <Ph_S1_, <Ph_T1_, AL_S1:T1_>>;*
*2.*  *Therefore Ph can be defined as follows: Ph = <Ph_S_, <<Ph_S1_, <Ph_T1_, AL_S1:T1_>>, AL_S:CT_>>, where Ph_S_ is a prefix of Ph_S1_, which means if Ph_S_ has n words, then Ph_S1_ has at least m + 1 words.*
*3.*  *Ph can be defined further as: Ph = <Ph_S_, << Ph_S1_, << Ph_S2_, <Ph_T2_, AL_S1:T1_ >>, AL_S1:T1_ >>, AL_S: CT_ >>, and so on.*

**Definition** **4.** *A Corpus-Trie:*

*1.*  *A parallel corpus C is a set of translated Phrases Ph;*
*2.*  *A Corpus-Trie CT is a trie representation of C. therefore CT is a trie;*
*3.*  *A Corpus-Trie CT is built using Definition 3 recursively from phrases;*
*4.*  *Since each phrase Ph (from Lemma 2) is a trie, the Corpus-Trie CT is a Trie;*

**Definition** **5.** *The Z-tree:*

*A Z-tree is a tree in the third dimension; its root is a node in the Ph-trie of the phrase Ph_S_ in the source language, and it has only one level, in which leaf nodes are several translated phrases Ph_T_ from the target language with their frequencies in the target corpus.*

**Lemma** **2.** *A phrase Ph is a prefix, trie-like, and only branches one-sided (left-sided); the whole phrase Ph is at the only leaf node of the trie.*

**Proof** **of** **Part 1.** Since *Ph* can be defined as follows:

*Ph* = <*Ph*_S_, <<*Ph*_S1_, <<*Ph*_S2_, <*Ph*_T2_, *AL*_S1:T1_>>, *AL*_S1:T1_>>, *Ph*_T_, *AL*_S:CT_>>, disregard at this point the translation of *Ph* (the phrase *Ph*_TS_ and its word alignment).

1. The trivial case: Let *Ph* have a single word *w*, i.e., *Ph = <Ph_S_>*, *and Ph_S_ = w*. Then *Ph* can be defined as a trie with one node *n* representing *w*;
2. Let *Ph* contain two words *w1* and *w2*. Then, it is defined as a trie of two nodes *n1* and *n2*, where node *n1* contains *w1* and node *n2* contains *w1* and *w2* (for a true trie, node *n2* will contain word *w2* only);
3. For the last case, *Ph*, which contains *m* words, can be represented by a trie-like representation of *m* nodes; the root will contain *w1*, the second node will contain *w1*, *w2*, and so on, until the *m*-th node, which will contain words *w1*, *w2*, *…*, *wm*. □

**Proof:** **of** **Part 2.** In the first part, we proved that the last node *m* would contain all the words in *Ph* as ordered. Therefore, the last node will contain *Ph*. □

**Definition** **6.** *Phrase Ph, including Ph_S_ and the corresponding Ph_T_, AL_S:CT_*

*Ph = <Ph_S_, <<Ph_S1_, <<Ph_S2_, <Ph_T2_, AL_S1:T1_>>, AL_S1:T1_>>, Ph_T_, AL_S:CT_>>*

*1.*  *The trivial case:*
*Let Ph contain only one word and its translation. Assume that the alignment is null; there is no alignment because it is a single word in the source language and one translated word in the target language, i.e., Ph = <Ph_S_, Ph_T_>. Ph has a one-to-one relation to Ph_S_, but Ph has a one-to-many relationship to Ph_T_, because the same word can be translated to one or more words in the target language. Ph* → *Ph_S_ where the arrow notation* → *is used to define one-to-one relationships, and the double arrow* →→ *is used to define one-to-many relations. The definition will be summarized using the arrow notation as follows:*
*Ph* → *Ph_S_* →→ *Ph_T_* → *AL_S:CT_;*
*Then, Ph can be represented as a trie with one node n representing Ph_S_ = w, and a tree in the the Z-dimension, with nodes that include the sets of ordered pair S_Z_ = (<Ph_T_, AL_S:CT_ >). One node, representing one of the elements of the set S_Z_, is labeled with the frequency of this Ph_T_ in the target corpus T and translates Ph_S_;*
*2.*  *Let Ph contain two words w1 and w2; then it can be represented by a trie (Ph-trie) of two nodes n1 and n2. Node n1 contains w1 and node n2 contains the ordered tuple <w1, w2>. In the Z-dimension, each node in Ph-trie will be a root node to a Z-tree including the different translations of the content of node X, in the target language with their frequencies, a set of ordered tuple S_Z_ = <X_T_, AL_S:CT_, F> where F represents the frequency of X_T_ in T and translates to X;*
*3.*  *The general case Ph which contains m words can be represented by a trie-like structure of m nodes. The first node contains w1, the second node contains w1, w2 and so on until the m-th node which contains words w1, w2,…., wm. Moreover, a Z-tree, in the third dimension, is built for all nodes.*

Figure 4a,b depicts an example clarifying Definition 6, for a phrase *Ph* of four words *<I*, *love*, *you*, *too>*. *Ti* represents a translation of word *wi* with frequency *F*.

## 5. Experimental Results

Experiments emphasized the cost of computation of the proposed method and whether translation request answering is adequately fast. The goal was to establish if the construction of the Corpus-Trie is affordable (though it is done offline). It was demonstrated that the translation request processing is fast and that Corpus-Tries have *O* (*log N*). *N* is noted as the count of phrases of the source language (excluding repetition); this is because the values of the nodes in the trie are sorted in each horizontal level.

### 5.1. Experimental Data

The United Nations Parallel Corpus v1.0 is composed of official records and other parliamentary documents of the United Nations, which are in the public domain. These documents are generally available in the six official languages of the United Nations. The corpus includes sentence-level alignments and allows access to multilingual corpora in various natural languages. We used the English-Arabic parallel corpus presented in [44]. It contains 456,552,223 pairwise-aligned English-Arabic sentences. We used two million of those pairs for our experiments. Building the general Corpus-Trie was done offline. Algorithm 1 was used to create Corpus-Tries from the sentences chosen from the bilingual corpus. We built nine different Corpus-Tries using 200,000 sentence pairs, with an increment of 200,000 sentence pairs for the next trie.

We also used the English-French parallel corpus presented in [44]. We used 500,000 pairwise-aligned English-French sentence pairs for our second set of experiments. Building the general Corpus-Trie was done offline. Algorithm 1 was used to create Corpus-Tries from the sentences chosen from the bilingual corpus. We built five different Corpus-Tries using 100,000 sentence pairs, with an increment of 100,000 sentence pairs for the next trie.

### 5.2. Building a Corpus-Trie

**Figure 5:**
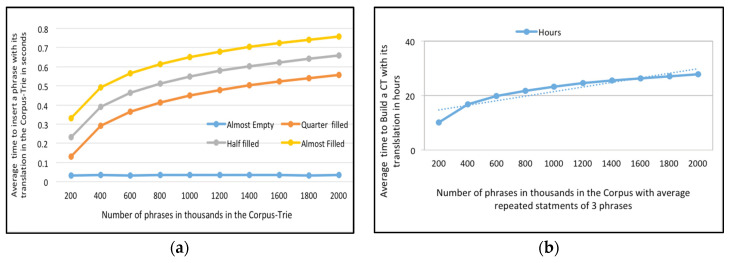
(**a**) Average time to insert a phrase with its translation in the Corpus-Trie in seconds versus Number of phrases in thousands in the Corpus-Trie for the English-Arabic parallel corpora; (**b**) The cost of *Corpus-Trie* construction (the number of phrases which are repeated in the corpus is in the average of three phrases), for the English-Arabic parallel corpora.

Having proven that Corpus-Trie aids fast translation-request processing, we wanted to establish that the time required to construct a CT from a database-phrase bilingual corpus is reasonable (see Figure 5a).

Lemma 1 established that the cost of inserting *N* phrases has an upper bound of *O* (*N Logn*). We measured the CPU time that Algorithm 1 required to convert a corpus-phrase bilingual corpus into a Corpus-Trie. Inserting a phrase into the Corpus-Trie required two steps, the first one being to search for the phrase, and the second step being dependent on the first step: either the phrase was not found, or the whole phrase or a portion of it was found. If the whole phrase was found, it did not have to be inserted, otherwise one or more nodes had to be created to insert it (see Figure 5b).

**Figure 6:**
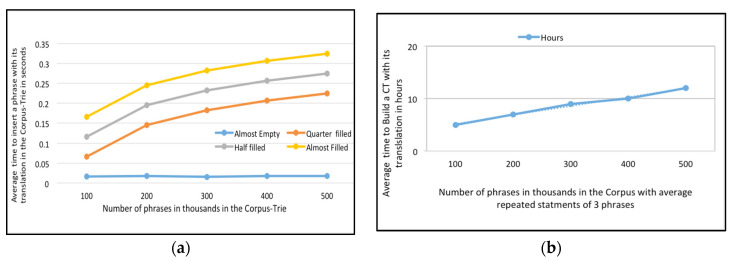
(**a**) Average time to insert a phrase with its translation in the *Corpus-Trie* in seconds versus Number of phrases in thousands in the *Corpus-Trie* for the English-French parallel corpora; (**b**) The cost of *Corpus-Trie* construction (the number of phrases which are repeated in the corpus is in the average of three phrases) for the English-French parallel corpora.

In Figure 6a, we measured the CPU time that Algorithm 1 requested to convert a corpus-phrase English-French corpus into a Corpus-Trie. In Figure 6b, we present the cost of Corpus-Trie construction (the number of phrases which are repeated in the corpus is an average of three) for the English-French parallel corpus.

### 5.3. Translation Request

In this subsection we introduce two types of experiments: the first type is the computation of the average cost of answering a translation request using different sizes of test data and corpus tries. The second type is calculating the average error rate of the translation process. The test data and the experiments are described in the following subsections.

#### 5.3.1. Test Data

We carried two types of experiments, for type I: our test data for both English-Arabic and English-French translations consists of six sets of 6000 English sentences each. Each set contains sentences of length equal to five words, seven words, 10 words, 13 words, 16 words, and 18 words respectively. Each set contains 100% of the sentences from the Corpus Trie. For experiment of type II: each set contains 90% of the sentences from the Corpus Trie, and 10% of the sentences that do not exist in the Corpus Trie, but either as a whole sentence or as an ordered subset of an existing sentence.

#### 5.3.2. Experiment Type I: Cost of Answering a Translation Request

In this type of experiments, the computational costs of answering a translation request were computed as an average by the node count in the Corpus-Trie that Algorithm 3 has to visit.

**Figure 7:**
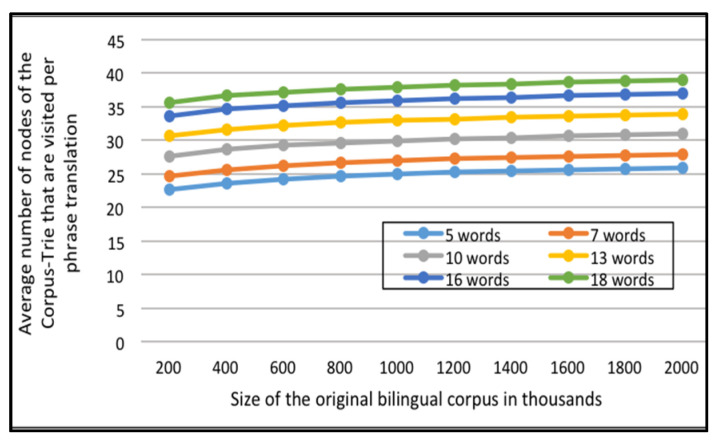
Average number of nodes of the *Corpus-Trie* that are visited per phrase translation versus Size of the original bilingual corpus in thousands, for the English-Arabic corpora.

The first set of experiments utilizes 1000 random translation requests for each set of the test data (All of them are presented in the portion of the English-Arabic corpus); The average number of nodes visited per translation request for each of ten Corpus tries, of different sizes, are computed as shown in Figure 7.

**Figure 8:**
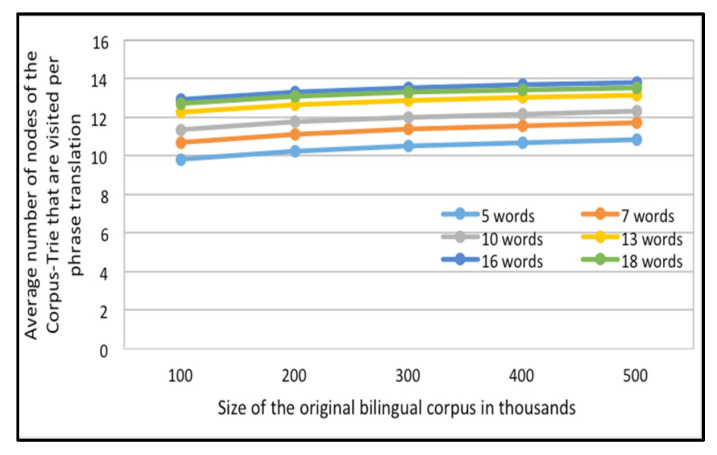
Average number of nodes of the Corpus-Trie that are visited per phrase translation versus Size of the original bilingual corpus in thousands, for the English-French corpora.

The second set of experiments utilizes 1000 random translation requests for each set of the test data (All of them are presented in the portion of the English-French corpus); The average number of nodes visited per translation request for each of ten Corpus tries, of different sizes, are computed as shown in Figure 8.

Figure 7 and Figure 8 contain multiple curves: one each for requests of phrases of five words and up, to requests containing phrases of 18 words, for the English-Arabic corpus and the English-French corpus, respectively.

It can be established that when answering a translation request, the system will navigate only a small part of the Corpus-Trie. The count of visited nodes is less than the log of the number of distinct original bilingual corpora.

#### 5.3.3. Experiment Type II: The Error Rate of The Translation Process

In this experiment, the error rate of the translation process is investigated for Corpus Trie of different sizes. We used 1000 phrases from each set that are randomly chosen from the data set, and we repeated the same experiment where 5000 phrases are randomly chosen for each set.

**Figure 9:**
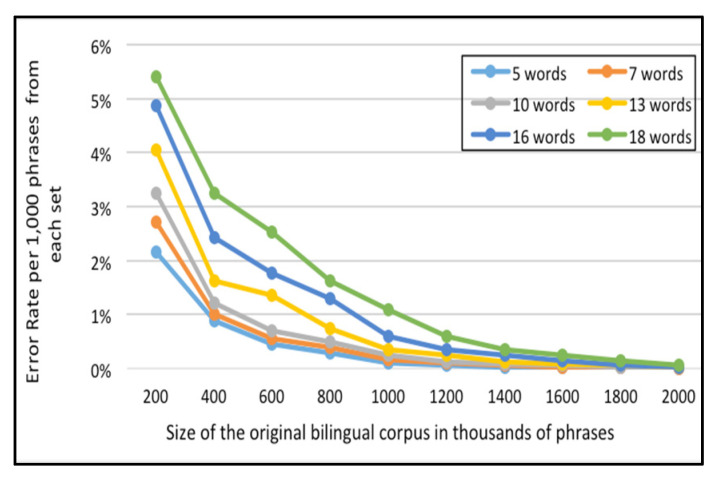
Error rate per 1000 phrases from each set (un-found phrases in the *Corpus-Trie*), for the English-Arabic corpora.

**Figure 10:**
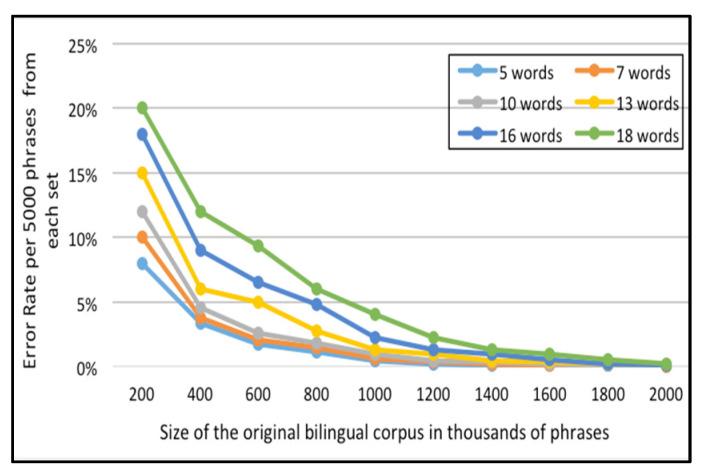
Error rate per 5000 phrases from each set, for the English-Arabic corpora.

For the English-Arabic corpus, Figure 9 and Figure 10 represent the error rate (i.e., the percentages of unfound phrases in the Corpus-Trie) per one thousand and five thousand phrases from each set respectively.

As indicated in Figure 9 and Figure 10, the percentage of failure to locate the phrase in the Corpus-Trie decreases with the increase of corpus size, and approaches zero with a corpus size of two million phrases.

**Figure 11:**
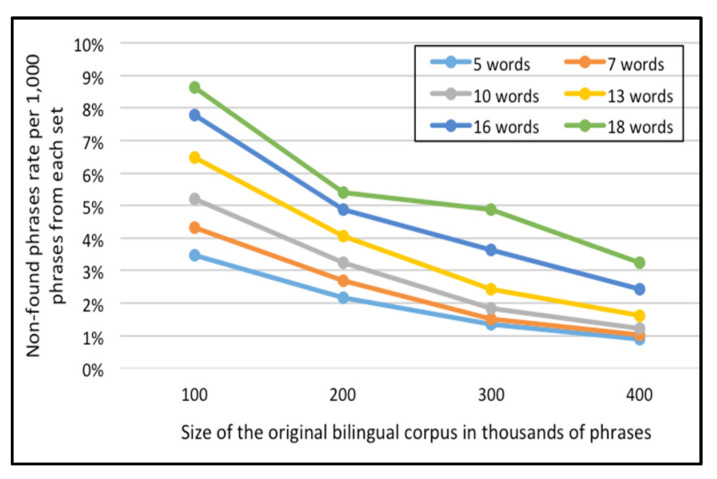
Error rate per 1000 phrases of each set, for the English-French corpora.

**Figure 12:**
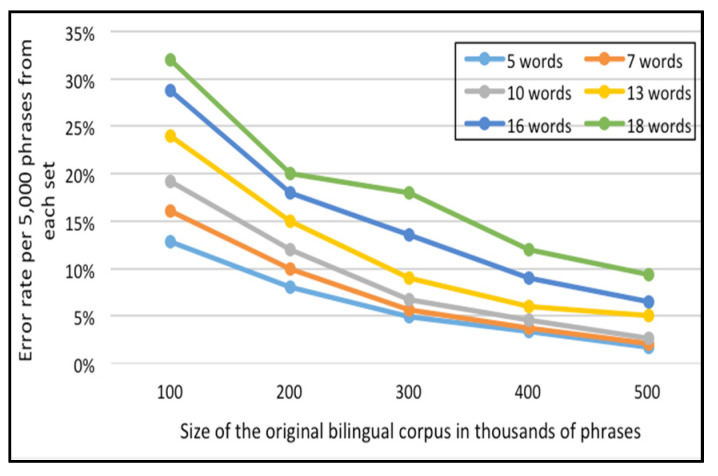
Error rate per 5000 phrases of each set, for the English-French corpora.

For the English-French corpus, Figure 11 and Figure 12 represent the error rate (i.e., the percentages of unfound phrases in the Corpus-Trie) per one thousand and five thousand phrases from each set of the test data respectively.

As indicated in Figure 11 and Figure 12, the percentage of failure to find the phrase in the Corpus-Trie decreases with the increase of corpus size, and approaches zero with a corpus size of 500 thousand phrases.

### 5.4. Translation Quality Evaluation

To assess the translation quality of our proposed system, we utilized manual and automated translation quality metrics. We compared our system against two open source machine translation platforms. The first one is Omega-T, which is an open source platform that utilizes different translation approaches [45]. In our comparison we used the property of translation memories (TMEM) reuse, which basically is the reusing of previous translations. Reference translations can also be included in TMEM from manual translations as well as from other machine translation systems. Also, same subject TMEM can be utilized such as translating legal document; previous translated legal documents can be reused. For our comparison we imported part of the data set from [44] as a TMEM in Omega-T platform.

The second platform is Apertium, which is an open source software for machine translation (MT) that is rule-based [46]. It is used to construct MT systems for a diversity of languages. Apertium utilizes linguistic facts gathered from different languages. It also utilizes multilingual dictionaries and grammatical rules of semantic and syntactic nature.

A qualitative evaluation of machine translation output is done both manually and automatically. Manual evaluation is done mainly by comparing translations from human experts to the output of machine translation, using human judges. The manual evaluation metrics of comparing our proposed system versus Omega-T and Apertium by human translators, are: fluency, adequacy, meaning, and preference.

We also include two other measures namely understandability and fidelity. Fidelity is a measure of the information retention in the translation text compared to the original one. While fidelity is measured with reference to both the original text and the translated text separately, understandability is measured with reference to the translated text only.

The human translation expert will first examine the translated sentence. The source sentence is then presented and judges would rate the original sentence on how more information they gained from it. The amount of information they gained from the original sentence is inversely proportion to the translation quality.

Automated quality evaluation of machine translation is performed using both BLEU and METEOR systems. BLEU is a very well-known MT quality evaluation and it estimates precision. METEOR is also well known but more complicated measure which estimates both precision and recall using F_mean_ score [47,48]. In the following subsections, we discussed the quality evaluation of our proposed system versus Omega-T and Apertium.

#### 5.4.1. Manual Evaluation of the Translation Quality

The translations from our proposed CT translation system were scored both manually and automatically. Three bilingual, native Arabic-speaking persons with master’s degrees or higher were asked to be volunteer evaluators. Each evaluator received an explanation of the scores. They made blind evaluations of three translation systems: System 1, System 2, and System 3, interchanged for each phrase translation. Omega-T [45], professional manual translation, and the CT system were compared. Each evaluator was asked to evaluate the same 100 phrase translations (20% were 7-word phrases, 20% were 10-word, 20% were 13-word, 20% were 16-word, and 20% were 18-word).

The evaluators were asked to evaluate phrases on Likert scales. They were asked to score four metrics: fluency, adequacy, meaning, and preference. Fluency was defined as an evaluation of readability ranging from 5 (perfect, “like reading an article”) to 1 (not understandable). Adequacy scores reflected evaluation of information conservation, ranging from 5 (100% information conservation) to 1 (0% information conservation). Meaning was defined as intent preservation, ranging from 5 (same meaning as the source phrase) to 1 (completely different meaning). The last measure was preference; an option was given to choose which translation was preferred using a two-answer scale of either 5 (strongly prefer) or 1 (do not prefer). Evaluators could give preference to one or more systems for each phrase translation. The results are presented in Table 1. The same experiment was carried out for the English to French translations; results are presented in Table 2.

#### 5.4.2. Automated Evaluation

Translation quality was also evaluated by an automatic process. Both BLEU and F_mean_ scores [47] were utilized. The BLEU score measures the precision of unigrams, up to four-grams, with respect to reference translations. BLEU measures accuracy, and takes values from zero to 100%; usually, a BLEU score of less than 15% implies bad translation, and a score of 50% is considered an excellent translation. The experiments were designed by comparing the average BLEU score of the proposed system against translations from Omega-T [45] and Apertium [46] translators. The results are shown in Table 3 and Table 4 for English-Arabic translation and English-French translation, respectively.

The results indicate that the BLEU score for the proposed CT system increases with the size of the number of phrases in the CT, for both English-Arabic and English-French translations. The proposed CT system was demonstrated to be better than both Omega-T and Apertium in quality of translation from a corpus size exceeding 1,600,000 phrases for English-Arabic translation, and 300,000 phrases for English-French translation.

Unlike BLEU, which only estimates precision, METEOR estimates precision and recall, and combines both using F_mean_ score [47,48]. Table 5 and Table 6 present automated evaluations using the F_mean_ score for the English-Arabic corpus and the English-French corpus respectively. Experiments were designed to compare the average F_mean_ score of the proposed system with translations from Omega-T and Apertium Translator. The results indicated that the F_mean_ metric for the proposed CT system increases with the size of the number of phrases in the CT for both English-Arabic and English-French corpora. For English-Arabic translation, the proposed CT system was shown to be superior to both Omega-T and Apertium in quality of translation from all corpus sizes beginning with 400,000 phrases, and to be dramatically enhanced by increasing the corpus size to two million phrases.

For English-French translation, the proposed CT system was demonstrated to be better than both Omega-T and Apertium in quality of translation for all corpus sizes. The results are shown in Table 5 and Table 6 for English-Arabic and English-French translations respectively.

### 5.5. Summary

Results of the experiments indicate that the computational cost required to process a translation request is logarithmic to the count of the distinct phrases in the bilingual corpus (and, thus the size of the Corpus-Trie). Only a small fraction of CT nodes (5% to 20% percent of the log of the number of the nodes) have to be visited. A Corpus-Trie of two hundred million phrases has a worst-case response time of 27.57542 nodes. Responding to the translation request using Apriori-based algorithms would be much more expensive.

### 5.6. Limitations and Future Extensions

We devised a qualitative assessment to track the limitations of our system to detect false negative, which means that the translation could be extracted from the bilingual corpora but was not done by our CT system.

We built a testing sample for our qualitative assessment, the sample consisted of 1000 phrases, 60% of the phrases are included in the source language of the CT, 10% of the phrases are included partially in the CT, while 10% of the phrases are included in the CT but as fragments not the whole phrases continuously. Another 10% of the phrases were included but with synonyms of some of the words. The last 10 % of the phrases are not included at all.

The qualitative assessment is summarized by showing example of true positive and false negative in Table 7. We used English to Arabic CT system as we are fluent in both languages.

In our system, we don’t have the notion of false negative as it only translates sentences that are presented in the Corus Trie either as a whole sentence or as ordered subset of a source sentence in the Corpus Trie. Therefore, we can conclude from the qualitative assessment that one of the limitations of our system is that we have no mechanism to union translations for phrases fragments that are already included in our corpus. A minor limitation is the lack of synonyms in the phrases of the source language, which can be included easily.

## 6. Conclusions

In this paper, we have introduced new concepts in machine translation paradigms, examining a bilingual corpus by submitting a translation request including the phrase *S* in the source language. We treated the corpus as a database of frequent word sets. We proposed a data structure called a Corpus-Trie that compresses a bilingual parallel corpus into a compact data structure representing a frequent data items set. We presented all required algorithms using the trie to answer translation requests, with novel properties and exhaustive experiments. Experiments were performed on English-to-Arabic and English-to-French translations, although the proposed method is not restricted to any specific language. Moreover, the proposed Corpus-Trie can be extended from a bilingual corpus to accommodate multi-language corpora in future iterations. We included the following algorithms that implement the following:

- Building a phrase trie with translation, alignment and frequencies;
- Z-tree insertion: inserting a translation, alignment, and update frequency if available;
- Building a Corpus-Trie from several phrase tries;
- Inserting a new phrase in the Corpus-Trie;
- Searching for a phrase in the Corpus-Trie and retrieving its translation.

Future extensions will include:

- Generalization to multi-language translation, enabling a source language to have translations from multiple target languages in the same Corpus-Trie;
- Inverted structure of the Corpus-Trie to benefit two-way translation;
- Compact implementation of Corpus-Trie structure.

## Figures and Tables

**Table 1:** Manual evaluation of the translation quality, for the English-Arabic corpora.

Score	Translation System		
	Omega-T	Professional Translation	The Proposed CT Translation System
Fluency	4.35	4.8	4.5
Adequacy	4.5	5	4.6
Meaning	4.3	5	4.4
Preference	4.1	5	4.5
Understandability	4.2	5	4.7
Fidelity	3	0	0.8

**Table 2:** Manual evaluation of the translation quality, for the English-French corpora.

Score	Translation System		
	Omega-T	Professional Translation	The Proposed CT Translation System
Fluency	4.01	4.8	4.4
Adequacy	4.2	5	4.45
Meaning	4.2	5	4.36
Preference	4.0	5	4.35
Understandability	3.8	5	4.5
Fidelity	3.4	0.3	0.7

**Table 3:** Automated evaluation using BLEU Score, for the English-Arabic corpora.

Translation System	Average BLEU Score		
	1000 Phrases	5000 Phrases	10,000 Phrases
Omega-T	41	42	45
Apertium Translator	41	43	44
CT translator (400,000 phrases)	39	40	39.5
CT translator (800,000 phrases)	42	43	43.5
CT translator (1,200,000 phrases)	44	45	46.3
CT translator (1,600,000 phrases)	46	46.7	46.5
CT translator (2,000,000 phrases)	49.2	49.9	51

**Table 4:** Automated evaluation using BLEU Score, for the English-French corpora.

Translation System	Average BLEU Score		
	1000 Phrases	5000 Phrases	10,000 Phrases
Omega-T	38	38.7	42
Apertium Translator	39	39.9	42.3
CT translator (100,000 phrases)	38.5	39	39.8
CT translator (300,000 phrases)	39	39.5	40.5
CT translator (500,000 phrases)	41	42	42.3

**Table 5:** Automated evaluation using F_mean_ Score, for the English-Arabic corpora.

Translation System	Average BLEU Score		
	1000 Phrases	5000 Phrases	10,000 Phrases
Omega-T	29	29.5	29.7
Apertium Translator	29.2	29.9	30
CT translator (400,000 phrases)	33	34	34.5
CT translator (800,000 phrases)	34	35	35.8
CT translator (1,200,000 phrases)	36	36.3	36.7
CT translator (1,600,000 phrases)	40	41.2	43
CT translator (2,000,000 phrases)	42	43.2	44.4

**Table 6:** Automated evaluation using F_mean_ Score, for the English-French corpora.

Translation System	Average BLEU Score		
	1000 Phrases	5000 Phrases	10,000 Phrases
Omega-T	31.5	32.3	34
Apertium Translator	33.7	33.8	34.3
CT translator (100,000 phrases)	34.3	34.7	35
CT translator (300,000 phrases)	35.2	35.1	36.3
CT translator (500,000 phrases)	37	37.4	38

**Table 7:** The qualitative assessment.

Phrases	CT System Output	Comment
Phrases are included fully in the CT	الحول هو حالة طبية تُعرّف على أنها نقص التنسيق بين العينين.	Good translation
“Strabismus is a medical condition that is defined as the lack of coordination between the eyes”.		
Phrases are included partially in the CT	No translation is Found	Although the following phrases are found in the CT but fragmented at different nodes and not included all in one phrase trie: When Strabismus is detected; at an older age; the chances of curing it; are slimmer.
“When Strabismus is detected at an older age, the chances of curing it are slimmer”.		
Phrases are included but with different synonyms in the CT	No translation is Found	We got no translation because of synonyms not included in the CT
“Strabismus is a medical condition that is known as the lack of coordination between the eyes”.		

## Data Availability

Data sharing not applicable.
